# Sweet potato extract alleviates high-fat-diet-induced obesity in C57BL/6J mice, but not by inhibiting pancreatic lipases

**DOI:** 10.3389/fnut.2022.1016020

**Published:** 2022-11-24

**Authors:** Tiange Liu, Fan Wu, Kejing Chen, Bingna Pan, Xifeng Yin, Yilin You, Zhixuan Song, Dan Li, Dejian Huang

**Affiliations:** ^1^National University of Singapore (Suzhou) Research Institute, Suzhou, China; ^2^Suzhou Kosmode Biotechnology Company, Suzhou, China; ^3^College of Food Science and Nutritional Engineering, Beijing Key Laboratory of Viticulture and Enology, China Agricultural University, Beijing, China; ^4^Department of Food Science and Technology, National University of Singapore, Singapore, Singapore

**Keywords:** sweet potato extract, resin glycoside, obesity, lipases, orlistat, hepatic steatosis

## Abstract

**Scope and aim:**

Sweet potato is widely consumed as a healthy and nutritive vegetable containing bioactive constituents for health promotion. This study investigated the beneficial impact of white-fleshed sweet potato extract (SPE) on high fat diet (HFD)-induced obese mice.

**Methods and results:**

First, SPE, in which resin glycoside was found as the dominant constituent, was suggested as a potential anti-obesity agent, because 20–70% pancreatic lipase (PL) inhibition was measured with SPE by *in vitro* turbidity assay and pNPP assay. Hence, next, the effect of SPE on obese mice was detected by oral administration of HFD supplemented with 6% SPE on C57BL/6J mice for 9 weeks. Surprisingly, being the opposite of what was typically observed from a lipase inhibitor such as orlistat, the fecal fat content in SPE-fed obese mice was decreased (*p* < 0.01). Meanwhile, 6% SPE supplement indeed significantly ameliorated HFD-induced obesity in mice, including body weight gain, fat accumulation, adipocyte enlargement, insulin resistance, and hepatic steatosis (*p* < 0.05). The improved liver steatosis was found associated with a down-regulating action of SPE on nuclear factor kappa B activation in HFD-fed mice. The anti-obesity influence of SPE was also confirmed on the HepG2 cell model for non-alcoholic fatty liver disease (NAFLD).

**Conclusion:**

These results indicate that SPE, as a dietary supplement, has the great potential for weight control and treating hepatic steatosis, possibly through a different action mechanism from that of orlistat.

## Introduction

During the last few decades, the global prevalence of obesity has been increasing (1). Moreover, its contribution to numerous chronic diseases, such as non-alcoholic fatty liver disease (NAFLD), type 2 diabetes mellitus, and cardiovascular diseases, makes obesity one of the main public health concerns (2). Intensive lifestyle intervention including strict dietary restriction and exercise is one of the primary means of adiposis control. However, the poor patient compliance often results in failure of long-term weight loss (3, 4). Anti-obesity drug (such as orlistat) therapy has been suggested for quick effect. Nevertheless, serious side effects caused by existing drugs have been reported, including stomachache, insomnia, heart attack, vomiting, headache, and constipation (5). Therefore, scientific researchers are searching for natural substances with anti-obesity bioactivity.

Sweet potato (*Ipomoea batatas* L.), as one of the main crops worldwide, possesses high nutrition value but also medicinal properties (6, 7). The tubers are delicious staple food for the world people, and can provide rich carbohydrates, vitamins, dietary fiber, and minerals. To date, the therapeutic effects of sweet potato tuber or leaf on hyperglycemia in type 2 diabetes mellitus, dyslipidemia, cardiovascular health, and inflammation have been reported (6–9), suggesting that sweet potato products can possibly be utilized for anti-obesity treatment. We found previously that resin glycosides (RG) exhibited potent pancreatic lipase (PL) inhibitory activity and RG is the active compound in the ethanolic extract of *I. batatas* leaves and tubers (10–12). Inspired by orlistat, which is an anti-obesity drug due to its inhibition effect on gastric and PL, sweet potato extract (SPE) containing RG has the great potential for weight loss in obese individuals. Therefore, the present study aimed to evaluate the weight loss effect of sweet potato tuber extract (SPE) in mice. Ethanolic SPE was prepared for *in vitro* PL inhibition assay at the beginning and next a high-fat-diet (HFD) induced mouse model. Orlistat was compared with SPE in this mouse study. C57BL/6J mice were fed HFD mixed with 6% SPE or 0.008% orlistat *ad libitum* for 9 weeks. The mouse body weight, adipose tissues at different locations of body, insulin sensitivity, and fecal lipids were measured after SPE or orlistat treatment. Liver was particularly evaluated for the lipid content and expression levels of inflammatory genes. NAFLD cell model was also employed to confirm the protective effect of SPE on HepG2 cells from fatty degeneration.

## Materials and methods

### Preparation of sweet potato extract

The dried powder of white-fleshed sweet potato (*I. batatas*) tuber was obtained from Zhangyiqing Agricultural Products Company (Linyi, Shandong, China). *I. batatas* tuber powder was mixed with 99% ethanol (1:3 w/w) and heated to 60°C for 3 h with stirring. The slurry was centrifuged and the supernatant was concentrated under reduced pressure to remove volatiles. The residue was washed with 99% ethanol in a solvent-to-solid ratio of 1:1 (w/w) for three times to remove starchy residues. The ethanol fractions were combined, dried under reduced pressure, to give *I. batatas* extract with 0.65% (w/w) yield based on the raw material powder. The extract was stored in freezer (–20°C) for future use.

### Measurement of total carbohydrates, reducing sugar, total phenolic, and resin glycoside content

The total carbohydrates content was determined using phenol-sulfuric acid method, according to the procedure of Nielsen (13). To each tube containing 2.0 ml of sample, 0.05 ml of 80% (w/w) aqueous phenol solution was added followed by 5.0 ml of concentrated sulfuric acid. After mixing on a vortex mixer, the test tubes were left at room temperature for 20 min. The absorbance was measured at 490 nm. The total carbohydrates content was calculated from the calibration curve of standard glucose solutions (0–50 μg/ml).

The reducing sugar content was determined using the 3,5-dinitrosalicylic acid method, according to the procedure of Saqib and Whitney (14) with modifications. DNS reagent was prepared by adding 1 g of DNS and 30 g of sodium potassium tartaric acid to 20 ml of 2 N NaOH. The solution was heated to 60°C with stirring until complete dissolution of reagents and then cooled down to room temperature followed by dilution with deionized water to 100 ml. For the measurement, 1.0 mL of DNS reagent was added to 0.5 ml of sample, and test tubes were kept at 95°C for 10 min using a hot water bath. The absorbance was measured at 540 nm after cooling down at room temperature for 10 min. The reducing sugar content was calculated from the calibration curve of standard glucose solutions (0–500 μg/ml).

The total phenolic content was determined using the Folin–Ciocalteu reagent, according to the procedure of Singleton and Rossi (15) with modifications. The Folin–Ciocalteu reagent was diluted to 10% (v/v) with deionized water before the test. On a 96-well microplate, 20 μl of sample was added to 100 μl of 10% Folin–Ciocalteu reagent, followed by 80 μl of 7.5% (w/v) sodium carbonate solution. The solution was incubated at 37°C for 30 min and the absorbance was measured at 765 nm. The total phenolic content was calculated from the calibration curve of standard gallic acid (0–100 mg/ml).

The total RG content was approximately determined using the silica gel chromatography. The crude extract was eluted with 100% hexane, 50% (v/v) hexane/ethyl acetate, 100% ethyl acetate, and 100% methanol, respectively. The resin glycoside fraction was collected, dried, and the yield was calculated according to the dry weight of crude extract and resin glycoside fraction.

### Pancreatic lipase inhibitory activity assay

All materials were purchased from Sigma (St. Louis, MO, USA) unless otherwise indicated. Olive oil was purchased from local supermarket and purified through neutral Al_2_O_3_ column to remove fatty acids. The purified olive oil was diluted in acetone by 0.33% (v/v) for better dispersion in buffer solution. Buffer solution was prepared by adding 0.35% (m/v) sodium deoxycholate into a 50 mM tris solution, and the pH was adjusted to 8.3 with 1.0 M HCl.

The turbidity assay used olive oil as lipase substrate and the procedure was obtained from literature with modifications (16). Oil emulsion was prepared by adding 200 μL of olive oil solution (0.33% v/v in acetone) into 1 mL buffer solution and sonicating on water bath at 70°C for 10 min. After cooling to room temperature, 5 μL of sample and 10 μL of PL solution (10 mg/mL) were added into 160 μL emulsion in the microplate well and the absorbance was measured at 590 nm every 30 s for 1 h at 37°C.

The pNPP assay used 4-nitrophenyl palmitate as lipase substrate. On a 96-well microplate, 5 μl of sample and 10 μl of PL solution (10 mg/ml) were added into 160 μl of buffer solution and incubated at 37°C for 10 min. The reaction started by adding 10 μl of pNPP solution (1.5 mg/ml in isopropanol) and the absorbance was measured at 410 nm every 30 s for 1 h at 37°C.

The tendency of readings in the first 5 min was regarded as linear growth, and the slope was calculated by linear regression. The inhibitory activity of each sample was calculated in percentage inhibition by the following Equation 1.


(1) Inhibition(%)=1-(slope/sampleslope)control×100%

### Animals and dosage regimen

Male C57BL/6J mice, 4 weeks old, purchased from the Vital River Laboratory Animal Technology Co Ltd. (Beijing, China), were housed in makrolon cages under standard laboratory conditions (light and dark cycle of 12 h at temperature 24 ± 2°C with a relative humidity of 55 ± 10%) with access to a normal chow diet (ND) and water *ad libitum* during 1 week acclimatization period. They were then randomly allocated to five groups of five mice each. The five groups were each allocated to different diets for 9 weeks as follows: ND (# AIN-93G, Dyets; 15.8% of calories derived from fat), HFD (D12492, Dyets; 60% of calories derived from fat), HFD + 6% SPE, ND + 6% SPE, and HFD + 0.008% orlistat. The dosages of SPE and orlistat for HFD-fed mice were equivalent to 573 and 0.89 mg/kg body weight in human, respectively. Detailed information on the diets is presented in Supplementary Table 1.

Body weights (BW) and food intakes were measured twice a week during the study. At the end of the study, mice were fasted for 16 h before sacrifice. The liver, epididymal white adipose tissue (eWAT), visceral adipose tissue (VAT), and subcutaneous white adipose tissue (sWAT) of each mouse were immediately removed and weighed. The blood was collected and biochemical markers including total cholesterol (TC), high density lipoprotein (HDL), low-density lipoprotein (LDL), and triglyceride in serum were measured by the VRL Laboratories China. All procedures of mouse experiments were approved by the Institutional Animal Care and Use Committee at Suzhou Institute of Systems Medicine (Suzhou, China) (ISM-IACUC-0048-R).

### Glucose tolerance test and insulin tolerance test

In the 6th week of the mouse study, mice were fasted for 16 h and then weighted. Blood was taken from the tail vein for measurement of initial blood glucose level using a glucometer (Roche, Shanghai, China). After that, mice were intraperitoneally injected with glucose (Beyotime, Shanghai, China) at a dose of 1.5 g/kg⋅ BW. Glucose was measured in whole blood from the tail vein at 0, 30, 60, 90, and 120 min using a glucometer.

Mice were fasted for 6 h before ITT. Blood was taken from the tail vein for measurement of initial blood glucose level. Mice were intraperitoneally injected with 1 IU/kg⋅ BW recombinant human insulin (Beyotime, Shanghai, China) and the glucose levels in whole blood from the tail vein were measured at 0, 30, 60, 90, and 120 min using Roche glucometer.

### Histological analysis of adipose tissue and liver

Liver and eWAT samples were fixed in 10% formalin overnight, dehydrated, paraffin embedded, and sectioned (6 μm sections) for H&E staining (Beyotime, Shanghai, China). For Oil Red O Staining, liver samples were embedded in Tissue-Tek O.C.T Compound (Sakura, Torrance, CA, USA) at room temperature for 30 min and then were stored at –80°C. Frozen samples (10 μm sections) were warmed up for 5–10 min before staining using Beyotime Oil Red O Staining Kit. Slides were imaged under optical microscope by ImageJ software for morphological observation.

### Fecal lipid extraction and measurement

Fresh feces from each mouse were collected twice a week during the last 3 weeks of the study. Lipids were extracted and measured according to the procedure of Jeejeebhoy with modification (17). Briefly, the fresh feces samples were dried in a vacuum oven at 45°C for 48 h before adding 1.5 ml of distilled water and four drops of concentrated hydrochloric acid (12 M). After 30 s of vortex, 6 ml of heptane: diethyl ether: ethanol (1:1:1, v:v:v ratio) were mixed with the samples, followed with 20 min incubation at room temperature. Next, the mixture was centrifuged for 15 min at 1,500 g before 3 mL of extraction solvent (made of heptane: diethyl ether: ethanol: water with1:1:1:1, v:v:v:v ratio) was added to the upper organic phase. The pellet was fully vortexed, agitated for 20 min at room temperature, and then centrifuged for 10 min at 1,500 *g*. The supernatant was removed and samples were placed in a fume cupboard for slow evaporation at room temperature, and then dried at 85°C under vacuum for 48 h. Samples were cooled down at room temperature before weighing. The content of fecal lipid was shown as mg/100 mg of dried feces.

### Cell culture and cell cytotoxicity test

The HepG2 cell line was purchased from the American Type Culture Collection (ATCC, Manassas, VA, USA). Cells were maintained in Minimum Essential Medium (Procell, Wuhan, Hubei, China), supplemented with 15% fetal bovine serum (Serana, Perth, WA, Australia) and 1% Penicillin-Streptomycin solution (Beyotime, Shanghai, China) at 37°C with 10% CO_2_. For cell cytotoxicity test, HepG2 cells at a concentration of 5 × 10^4^ cells per well were seeded in a 96-well microplate and allowed to adhere to the well. The medium was replaced with serum-free Minimum Essential Medium containing 1 mM free fatty acid (FFA) and 1% BSA, with the treatment of SPE (0/1/10/20/30/40/50 μg/ml) at 37°C for 24 h. Subsequently, the cells were incubated with 10 μl of CCK-8 (Beyotime, Shanghai, China) and serum-free Minimum Essential Medium at 37°C for 2 h. The experiment was performed in triplicate. The results were expressed as percentage of viable cells with respect to the untreated cells. The absorbance was measured at 450 nm using a microplate spectrophotometer system (BioTek, Shoreline, WA, USA).

### Determination of cell lipid and triglyceride contents

To induce excessive lipid accumulation in an *in vitro* NAFLD cell model, HepG2 cells were seeded at 5 × 10^4^ cells per well in a 96-wells plate, and divided into five groups: blank, FFA induction, and SPE treatment group (1, 10, and 20 μg/ml). FFA (1 mM) was prepared by dissolving oleic acid and palmitic acid (concentration ratio 2:1). HepG2 cells were firstly incubated with SPE solution at 37°C for 4 h, flowing with the incubation of FFA–BSA complex at 37°C for 24 h. Control cells were treated with 1% BSA only. HepG2 cells were washed with PBS, fixed in 4% paraformaldehyde for 10 min, and then stained with Oil Red O at room temperature (Beyotime, Shanghai, China) for 30 min. The stained cells were washed with distilled water before photographing. For lipid content quantification, the stained cells were incubated with isopropanol for 10 min at room temperature, and the absorbance at 500 nm was measured. The results were expressed as percentage of viable cells with respect to untreated control cells. To determine the synthesis of triglyceride, cells were seeded in 6-well plates, treated under the same conditions, harvested with absolute ethyl alcohol, and fully lysed by ultrasonicator treatment. After centrifugation, the supernatant was collected to measure the concentration of triglyceride according to the standard samples.

### Real-time-PCR analysis of mRNA expression

Total RNA was extracted from mouse livers or HepG2 cells using Trizol reagent (Tiangen, Beijing, China). One microgram of total mRNA was used for reverse transcription of cDNA using the RT-PCR Synthesis Kit, according to the manufacturer’s instructions (Biosharp, Suzhou, Jiangsu, China). The cDNA was performed on a 384-qPCR system using SYBR Premix (Roche, Suzhou, Jiangsu, China). The mRNA level of actin was relative to internal control. The expression of mRNA was calculated by 2^–ΔΔCT^ method. Detailed information of primers is shown in Supplementary Table 2.

### Statistical analysis

All the data were expressed as means ± standard error of mean (SEM). Statistical analysis was conducted using GraphPad Prism (version 8.0). The statistical differences between the groups were evaluated using Ordinary one-way ANOVA. *p* < 0.05 was considered statistically significant.

## Results

### Sweet potato extract inhibits pancreatic lipase activity *in vitro*

In the present study, we prepared SPE and found that SPE can inhibit PL activity in a dose-dependent manner. This is in agreement with our previous work (12). A PL inhibition of 20–70% was measured with SPE concentrations at 20–150 μg/ml by turbidity assay (Figures 1A,B) and 15–70 μg/ml by pNPP assay (Figures 1C,D). The apparent IC_50_ of the extract against PL using the two *in vitro* assays were 55.7 and 36.9 μg/ml, respectively.

**FIGURE 1:**
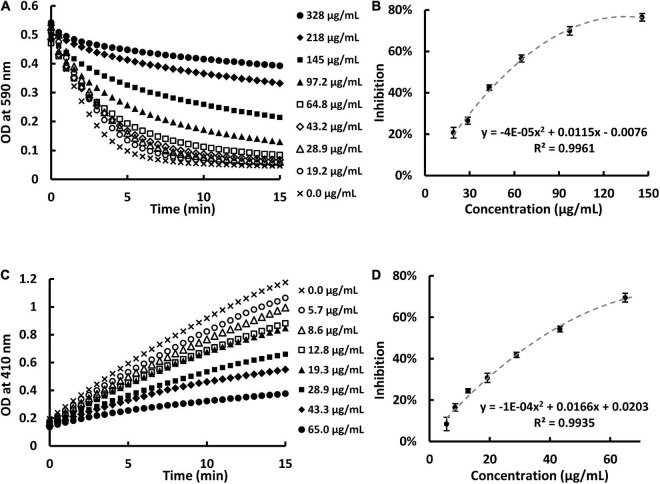
Sweet potato extract (SPE) inhibited pancreatic lipase (PL) activity *in vitro* in a dose-dependent manner. **(A)** Kinetic readings of optical density at 590 nm in the presence of eight concentrations of SPE using turbidity assay. **(B)** Dose-response curve of PL inhibition against concentration of SPE using turbidity assay. **(C)** Kinetic readings of optical density at 410 nm in the presence of seven concentrations of SPE using pNPP assay. **(D)** Dose-response curve of PL inhibition against concentration of SPE using pNPP assay.

We further analyzed the components of ethanolic SPE and found that it contains a good amount of total carbohydrates 230 ± 15 mg/g (glucose equivalent) but negligible reducing sugar content at 2.9 ± 0.5 mg/g (glucose equivalent). This result suggests that there is a significant amount of resin glucosides, which are non-reducing carbohydrates composed of esters of oligosaccharides with four or five monosugar units. Naturally occurring RG consist of multiple homologues sharing similar structure. HPLC and MS analysis of the resin glycoside are shown in Supplementary Figure 1 and Supplementary Table 3. Sweet potato has been reported to contain some polyphenolic compounds which might be responsible for bioactivity. Therefore, we quantified the total phenolics of the extracts and found that it has less than 2% (18.3 ± 1.2 mg/g, gallic acid equivalent). On the other hand, total resin glycoside content (560 ± 42 mg/g) was the dominant component in our extract. Inspired by orlistat, which is also a potent PL inhibitor, next, we designed a diet induced obesity mouse model to evaluate the anti-obesity potential of SPE *in vivo*.

### Sweet potato extract supplement protects mice against high fat diet-induced fat accumulation

**FIGURE 2:**
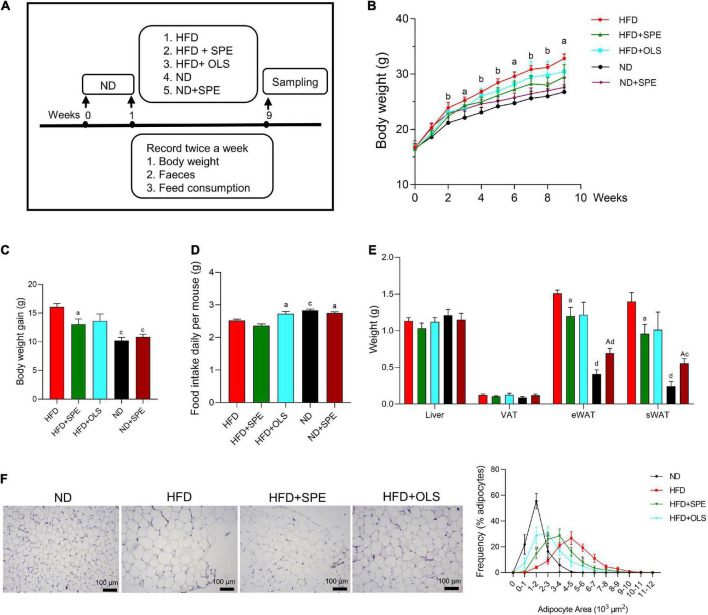
Sweet potato extract (SPE) ameliorated high fat diet (HFD)-induced body weight (BW) gain and fat accumulation. **(A)** The study design. **(B)** BW of mice in every group during the entire period of 9 weeks. **(C)** BW changes of mice in five groups. **(D)** Daily food intake of mice in five groups. **(E)** Weight of mouse liver and adipose tissues at endpoint. **(F)** Representative pictures of the adipose tissue in H&E staining and the size of adipocytes. Values are means ± SEM, *n* = 5. Results having different letters indicate statistical difference. In panel **(B)** the comparison between HFD group and the HFD + SPE group, “a”, *p* < 0.05; “b”, *p* < 0.01. In panels **(C–E)** the comparison with the HFD group, “a”, *p* < 0.05; “b”, *p* < 0.01; “c”, *p* < 0.001; “d”, *p* < 0.0001; the comparison with the normal diet (ND) group, “A”, *p* < 0.05.

The experimental design flowchart is shown in Figure 2A. Although ND mice exhibited a higher daily food intake as compared to HFD mice, the daily energy consumption of HFD mice was as high as 13.3 ± 0.2 kcals per mouse, much more than that of ND mice (*p* < 0.0001) (Figure 2D; Supplementary Table 1). Consequently, the mice fed with HFD displayed significantly increased BW (Figures 2B,C,E), eWAT, and sWAT than those fed with ND. At the endpoint, the BW gain in the HFD group was 16.07 g, around 1.58 times the BW gain in ND group. Consistently, the size of the majority of adipocytes in the fat bodies around the epididymis was 4–5 × 10^3^ μm^2^ in HFD fed mice and 1–2 × 10^3^ μm^2^ in ND fed mice as comparison. Moreover, no large adipocyte (>5 × 10^3^ μm^2^) was observed in the eWAT of ND control mice, while the percentage of large adipocytes (>5 × 10^3^ μm^2^) in the eWAT of HFD fed mice was 39% (Figure 2F). The histological analysis together with weekly BW data suggests that the administration of HFD for 9 weeks could induce obesity in C57BL/6J mice.

Since Week 2 the mice fed 6% SPE supplementation started showing significantly lower BW than HFD-fed mice (*p* < 0.01) and the remarkable anti-obesity effect remained till the end of study, which partly resulted from the weight loss of sWAT, eWAT, VAT, and liver, with a 31, 20, 16, and 9% reduction (Figures 2B,E). Again, consistently, the SPE supplementation also reduced the sizes of most adipocytes from 4–6 × 10^3^ μm^2^ (46% adipocytes in HFD-fed mice) to 2–4 × 10^3^ μm^2^ (54.3% adipocytes in SPE-fed mice). In addition, the proportion of large adipocytes (>5 × 10^3^ μm^2^) decreased markedly by 25% in SPE group as compared to HFD group (Figure 2F).

0.008% orlistat supplementation inhibited the weight gain and fat accumulation of HFD-fed mice as well but not significantly, possibly due to the increased food intake of HFD + OLS mice (Figure 2D). In contrast, the addition of SPE in either ND or HFD did not alter murine feeding behavior.

To take together, our results revealed that the treatment of C57BL/6J mice with 6% SPE supplementation can effectively promote weight loss by decreasing the formation of adipose tissues.

### Sweet potato extract supplement enhances insulin sensitivity but not glucose intolerance in high fat diet-fed mice

**FIGURE 3:**
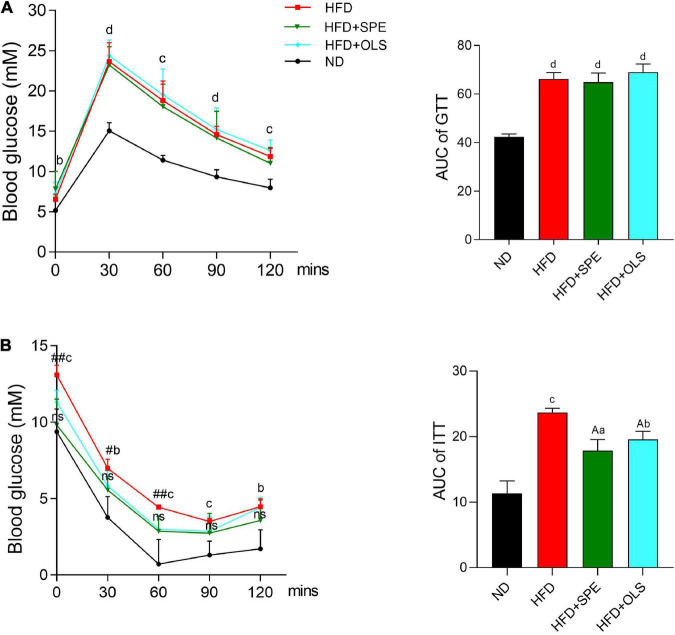
Sweet potato extract (SPE) improved insulin resistance but not glucose intolerance in high fat diet (HFD)-fed mice. **(A)** Blood glucose levels and area under curve (AUC) in glucose tolerance test (GTT). **(B)** Blood glucose levels and AUC in insulin tolerance test (ITT). Values are means ± SEM, *n* = 5. Results having different letters indicate statistical difference. In the comparison with the normal diet (ND) group, “a”, *p* < 0.05; “b”, *p* < 0.01; “c”, *p* < 0.001; “d”, *p* < 0.0001; “ns”, *p* > 0.05. In the comparison with the HFD group, “A”, *p* < 0.05. In the comparison between the HFD group and the HFD + SPE group, “#”, *p* < 0.05; “##”, *p* < 0.01.

Obesity is often associated with glucose intolerance and insulin resistance. Therefore, we performed GTT and ITT to compare the changes of glucose levels in the blood of mice fed with different diets. After administrating glucose solution, the levels of blood glucose in all the mouse groups increased rapidly and reached their peaks within 30 min, and then gradually declined in 1.5 h (Figure 3A). At all of five monitoring time-points, the glucose levels in the blood of HFD-related mouse groups remained significantly greater than that in the blood of ND fed mice. The tolerance against glucose estimated by GTT AUC has demonstrated that 9 weeks-feeding of HFD impaired glucose homeostasis and unfortunately, the supplement of neither SPE nor orlistat showed ameliorating effect.

Mice were fasted for 6 h before ITT. HFD-fed mice still displayed a significantly higher level of fasting glucose as compared to the control (*p* < 0.001) (Figure 3B). In contrast to HFD, the supplement of SPE restrained the fasting glucose in the HFD + SPE mice at a similar level as the ND mice, indicating the improved fasting glucose resulted from SPE supplement. During the 2 h monitoring period of ITT, HFD fed mice remained the highest level of blood glucose (*p* < 0.01) as compared to ND fed mice, while no significant difference of the glucose levels was found between the HFD + SPE group and the ND group. In line with the glucose changes of ITT, the AUC results also demonstrated that the treatment of SPE effectively enhanced insulin sensitivity in the HFD-induced obese mice.

### The action mechanism of sweet potato extract for weight control is different from that of orlistat

Orlistat was approved as a prescription product by FDA in 1999 for weight management. After treatment of orlistat in obese subjects, increased fecal fat is often presented, because orlistat’s action of mechanisms is the selective inhibition of gastrointestinal lipase activity (18), which was also discovered with the treatment of SPE in our *in vitro* PL inhibition assay. Therefore, we evaluated the lipid content in murine sera and feces to explore whether SPE could decrease the dietary fat absorption for fat loss in the HFD induced mouse model.

**FIGURE 4:**
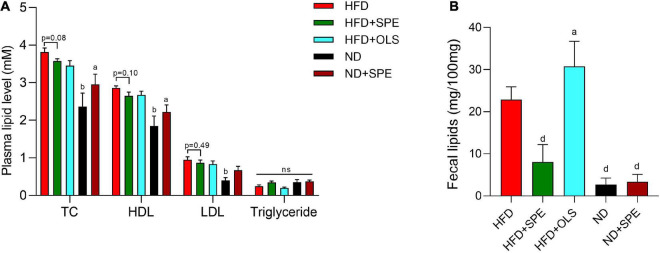
Sweet potato extract (SPE) decreased plasma cholesterol and fat excretion by fecal route. **(A)** The levels of total cholesterol (TC), high density lipoprotein (HDL), low-density lipoprotein (LDL), and triglyceride in mouse plasma. **(B)** The content of lipids in mouse feces. Values are means ± SEM, *n* = 5. Results having different letters indicate statistical difference. In the comparison with the high fat diet (HFD) group, “a”, *p* < 0.05; “b”, *p* < 0.01; “d”, *p* < 0.0001; “n.s”, not significant.

High fat diet-fed obese mice displayed higher levels of TC, HDL, and LDL than ND-fed control mice (*p* < 0.01). The levels of TC, HDL, and LDL in plasma were not reduced in a statistically significant manner (TC, *p* = 0.08; HDL, *p* = 0.10; LDL, *p* = 0.49) by the SPE supplement in HFD as compared to HFD-fed mice (Figure 4A). On the contrary, the supplement of SPE in ND seemed to slightly increase, albeit not statistically significantly, the levels of TC, HDL, and LDL in the plasma in comparison with ND-fed mice (Figure 4A). Consequently, the differences of TC, HDL, and LDL between ND + SPE group and HFD group was less significant than those between ND group and HFD group (*p* < 0.05 in TC and HDL, but not significant in LDL). In line with the previous reports of orlistat, the HFD-fed mice excreted a remarkably larger amount of fecal fat as compared to ND-fed mice (*p* < 0.0001), and the orlistat supplement in HFD greatly promoted more fat discharge (*p* < 0.05) (Figure 4B). In contrast, the SPE supplement significantly reduced the level of fecal lipids in HFD-fed mice (*p* < 0.0001), indicating a distinct mechanism of action for weight loss as compared to orlistat in our diet induced-obesity murine model.

### Sweet potato extract supplement alleviates hepatic steatosis by down-regulating nuclear factor kappa B activation in high fat diet-fed mice

**FIGURE 5:**
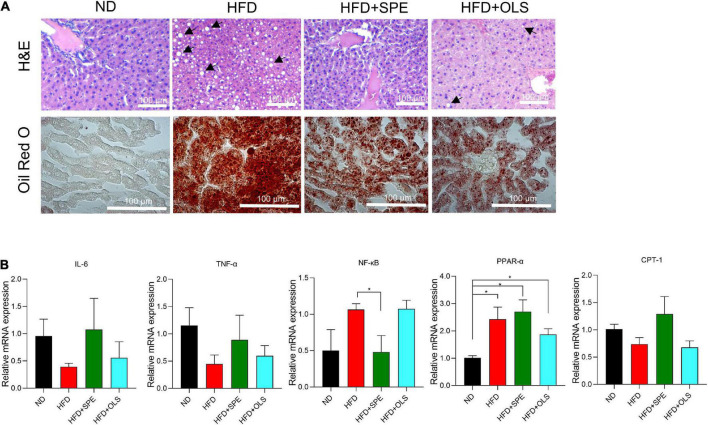
Sweet potato extract (SPE) suppressed nuclear factor kappa B (NF-κB) activation during liver steatosis, but inflammation was not caused in the high fat diet (HFD)-fed mice. **(A)** Representative pictures of the liver from every group of mice in H&E and Oil Red O staining. **(B)** Relative mRNA expression of NF-κB, IL-6, tumor necrosis factor α (TNF-α), peroxisome proliferator-activated receptor α (PPAR-α), and carnitine palmitoyl transferase 1 (CPT-1) in mouse livers. The black arrows indicate major hepatic macrovesicular steatosis. Values are means ± SEM, *n* = 5. *p < 0.05.

We conducted H&E and Oil Red O staining of mouse liver sections for assessing the degree of liver steatosis in HFD-fed mice. The histological analysis showed significant hepatocyte vacuolization and abundant lipid droplets in the liver of HFD-fed mice as compared with the ND-fed control mice. Notably, with the administration of SPE, the HFD caused liver steatosis was attenuated in C57BL/6J mice. To better understand the mechanism of liver lipogenesis regulation, the mRNA expression of nuclear factor kappa B (NF-κB), IL-6, tumor necrosis factor α (TNF-α), peroxisome proliferator-activated receptor α (PPAR-α), and carnitine palmitoyl transferase 1 (CPT-1) were determined by real-time PCR (Figure 5) and the results shown that, although 9 weeks-feeding of HFD did not cause an increase of inflammation-associated cytokine expression including IL-6 and TNF-α, the gene expression of PPAR-α in the livers of HFD mice was significantly up-regulated, which has been believed to decrease the triglyceride level and be involved in regulation of energy homeostasis (19). In our present study, the SPE supplement failed to change the expression of PPAR-α in a statistically significant manner (Figure 5B). Nevertheless, the mRNA expression of NF-κB was greatly restrained in SPE-fed mice as compared to HFD-fed mice. Compelling evidence has revealed an essential role of the NF-κB signaling pathway in the pathologies of steatosis related liver complications (20). The decline of NF-κB axis activation supported our histological results which showed the improvement of hepatic steatosis in SPE-fed mice. Moreover, CPT-1, as an influencing element in long-chain fatty acid oxidation and glucose production by the liver (21), seemed to be unaffected by HFD or HFD + SPE diet in mice.

### Sweet potato extract confers preventive effects against free fatty acid-induced hepatic lipid accumulation

**FIGURE 6:**
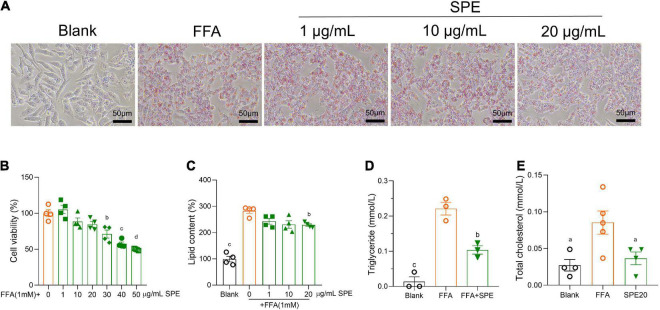
Sweet potato extract (SPE) inhibited free fatty acid (FFA) induced lipid accumulation in HepG2 cells. **(A)** Representative pictures of the HepG2 cells with different treatments in Oil Red O staining. **(B)** Cytotoxicity of SPE on HepG2 cells. **(C–E)** The effect of SPE on lipid content, triglyceride, and total cholesterol (TC) in FFA-induced HepG2 cells. Results having different letters indicate statistical difference. In the comparison with the FFA group, “a”, *p* < 0.05; “b”, *p* < 0.01; “c”, *p* < 0.001; “d”, *p* < 0.0001. Values are means ± standard error of mean (SEM), *n* = 4 in panels **(B,C,E)**, *n* = 3 in panel **(D)**. The data is representative of three independent experiments.

To further confirm the influence of SPE on lipid accumulation in livers, we built an FFA induced *in vitro* model of NAFLD using immortalized human cell lines HepG2. Firstly, a serial concentration of SPE was tested on HepG2 cells for cytotoxicity. As shown in Figures 6A,B, the cytotoxicity of SPE on HepG2 cells displayed a dose-dependent manner and became significant from 30 μg/ml. Therefore, 1–20 μg/ml SPE were chosen for incubation with HepG2 cells during FFA induction. As shown in Figure 6A, morphological observation demonstrated that adding FFAs led to a significant increase of lipid droplet accumulation in the cytoplasm which was evaluated qualitatively by Oil Red O staining. Also, after FFA induction the intracellular lipid content (%) in HepG2 was almost tripled (282.3 ± 9.17) as compared with the blank control (99.2 ± 10.73), demonstrated in Figure 6C. Likewise, the levels of triglyceride and TC in hepatocytes were remarkably promoted by FFAs in Figures 6D,E. The optimum efficacy of SPE was found at the concentration of 20 μg/ml, which lowered the levels of lipid content, triglyceride, and TC by 19, 53, and 57%, respectively.

## Discussion

The main objective of the present research was to determine the effects of SPE supplement on HFD-induced obesity in C57BL/6J mice. The obesity-associated disturbances in mice included the impairment of glucose homeostasis and lipid metabolism, enlarged adipocytes and hepatic steatosis, which were detected by measuring murine adipose tissue, liver, feces, and blood. The previous work from our lab found that the PL activity was inhibited notably by SPE as evidenced by *in vitro* assays (12). Since orlistat presents as a potent gastrointestinal lipase inhibitor against both pancreatic and gastric lipases (22), we proposed a scientific hypothesis that SPE could be used as a natural food supplement to promote weight control in obese individuals in a similar mechanism of action as orlistat. In the present study, HFD supplemented with 6% SPE was fed daily in C57BL/6J mice. Consequently, the *in vivo* beneficial effects of SPE presented especially significant on ameliorating BW, eWAT, sWAT, insulin sensitivity, and NF-κB associated fatty liver. However, the content of excretive fat through gastrointestinal system was not increased but even decreased due to SPE supplement, which was unexpectedly the contrary to the response to orlistat supplement in humans (22), suggesting alternative pathways to be further determined.

Sweet potato is a widely grown root crop worldwide and has increasing popularity because of its nutrition value and multiple health functions. Both sweet potato tubers and leaves have been considered as excellent novel sources of natural health-promoting compounds (23). For instance, caffeic acid derivatives (24, 25), chlorogenic acid (26, 27), caffeoylquinic acid derivatives (24, 28–30), anthocyanins (31), and quercetin (32) in sweet potato leaves have been found antidiabetic and anticancer. The extract of sweet potato tuber, especially that of the purple sweet potato tuber, has been recognized as an antioxidant agent due to the high content of polyphenols (33). Purple SPE has shown an inhibitory effect on oxidation of LDL or protein glycation as evidenced using THP-1 cell line and human plasma (34). Therefore, Park et al. concluded that the purple SPE has the essential potential treat hyperlipidemic and hyperglycemic disorders. Hyperglycemia, hyperlipidemia, and diabetes are all closely linked with obesity. Hence the anti-obesity components are likely to be discovered in SPE. Kim et al. prepared the crude extracts of anthocyanin and carotenoid from color-fleshed sweet potatoes and demonstrated that the treatment of these two major pigments were capable to diminish adipogenesis of the intermediate stage in HFD-induced C57BL/6N obese mice *in vivo* and over 63% of fat accumulation in 3T3-L1 adipocytes (35). Unlike purple-or orange-fleshed sweet potato, white-fleshed sweet potato contains little anthocyanin and carotenoids (36), and to date has not been studied for its anti-obesity potential. Our study presents the first direct demonstration that white-fleshed sweet potato exhibited significant anti-obesity activities under a probably different mechanism of action from orlistat, although RG contained in sweet potatoes were found capable to inhibit PL *in vitro*. Further research is warranted to elucidate the molecular mechanisms of RG for body weight control.

Obesity, defined as the accumulation of excessive body fat, has become a severe public health concern globally. The manifestations of obese patients include elevated BW, increased levels of TC, HDL, LDL, and glucose in circulating blood, enlarged adipose tissues at different body locations and metabolic dysregulation (37, 38), which have been presented in this HFD-induced obese mouse model. Excess calories cause high levels of glucose and FFAs, which force adipocytes to gradually accumulate more lipid and expand in size (37, 38). This study has evidenced that daily supplement of SPE led to a much lower level of blood glucose after 6 h fasting and apparently the smaller size of adipocytes in HFD-fed mice, and thus the eWAT, sWAT, and overall BW were significantly deceased. The augmentation of fat within the cytoplasm of hepatocytes results in hepatic steatosis, which is a common companion of obesity and a valuable predictor for reflecting the increments of whole-body fat mass (39, 40). Insulin resistance and impaired glucose homeostasis have been considered as essential features of obese patients with steatosis (39, 41). The manifestations of this HFD-induced mouse model well-mimicked the main symptoms of obese patients. HFD led to insulin resistance and severe lipid accumulation in the murine liver, which were effectively improved by the daily administration of resin glycoside enriched SPEs. However, the GTT results indicate that glucose tolerance of HFD-fed mice was not ameliorated by SPE supplement in week 6 of this study. This situation could be influenced by the feeding duration and GTT time-point, since prolonged treatment of HFD often confers more significant metabolic disorder and the regulatory effect of anti-obesity agents on glycemic control can be optimized by administration for an extended period (e.g., over 12 weeks) (42, 43). Likewise, there was no significant upregulation of IL-6 and TNF-α in mouse fatty livers of HFD-fed mice, which indicates the limited levels of inflammatory responses.

The aberrant activation of liver NF-κB pathway mediated by nuclear factor-κB-inducing kinase (NIK) has been reported previously in obese conditions (44, 45). In agreement with the results of NIK deletion or suppression strategies, in the present research the supplement of SPE in HFD successfully restored NF-κB expression levels to normal as compared to HFD-fed group, while the supplement of orlistat did not. Therefore, in contrast to orlistat, the efficacy of which for NAFLD and non-alcoholic steatohepatitis remains controversial (46, 47), SPE may become a superior option as supplementary ingredients for treating obesity-associated hepatic steatosis. The inhibited mRNA expression of NF-κB in SPE-fed mice also encouraged us to explore the role of PPAR, the ligands of which have anti-inflammatory activities by inhibiting multiple transcription factors such as NF-κB. As demonstrated in Figure 5B, the mRNA expression levels of PPAR-α were significantly higher in every group of HFD-fed mice than that in ND control, which supports the anti-inflammatory role of PPAR-α during the development of obesity. PPAR-α and CPT-1 have been evidenced to be involved in beta-oxidation of fatty acids to reduced lipid accumulation and the upregulation of them can improve glucose and lipid metabolism (19, 21). In the present research, although the fatty liver was remarkably ameliorated in SPE-fed mice, SPE had the tendency to promote the mRNA expression levels of PPAR-α and CPT-1, but did not reach a significant difference.

## Conclusion

In conclusion, the present study has elaborated that in the male C57BL/6J obese mice, SPE can alleviate the weight gain, adipose tissue expansion, insulin sensitivity, and lipotoxic injury in the liver. These findings may be useful for the treatment and prevention of obesity in humans. However, the mechanisms under the anti-obesity effect appear to be yet unclear. Whereas RG in SPE was once considered as the inhibitor of weight gain under an orlistat-like mechanism of action, the decreased content of fecal fat in SPE-treated mice as compared to HFD-fed mice denies this hypothesis. Rather, with the administration of SPE, the excessive fat appears to be less removed mechanically through gastrointestinal tract. Where did the excessive dietary fat go? According to a recent study in our team (48), the resin glycoside extract with high molecular weight is relatively stable when exposed to different digestive enzymes (e.g., alpha-amylase and pepsin) and the gastrointestinal pH. Therefore, the beneficial role that SPE, rich of resin glycoside, plays in obese individuals is suspected of being related with gut microbiota regulation. Conceivably, in the future, more investigations of fecal microbiota, dietary fat metabolism and energy expenditure can help to better understand the mechanism based on which SPE confers an improved utilization of excess calories leading to healthier conditions in obese individuals.

## Data availability statement

The original contributions presented in this study are included in the article/Supplementary material, further inquiries can be directed to the corresponding authors.

## Ethics statement

This animal study was reviewed and approved by the Institutional Animal Care and Use Committee at Suzhou Institute of Systems Medicine.

## Author contributions

TL conceived the study, designed the experiments, and wrote the manuscript. FW performed the experiments and contributed to the data analysis. KC performed the fecal lipid extraction and assisted in cell culture. BP performed the histological analysis of adipose tissue and liver. XY prepared the sweet potato extract. YY supervised the experiment design and reviewed the writing. ZS conceived the study and performed the pancreatic lipase inhibitory activity assays *in vitro*. DL supervised the writing revision. DH conceived the study and supervised the experiment design and writing revision. All authors contributed to the article and approved the submitted version.

## Abbreviations

AUC: area under curve
BW: body weight
CPT-1: carnitine palmitoyl transferase 1
eWAT: epididymal white adipose tissue
FFA: free fatty acid
GTT: glucose tolerance test
HDL: high density lipoprotein
HFD: high-fat diet
ITT: insulin tolerance test
LDL: low-density lipoprotein
NAFLD: non-alcoholic fatty liver disease
ND: normal diet
NF-κB: nuclear factor kappa B
OLS: orlistat
p-NPP: p-Nitrophenyl phosphate
PPAR: peroxisome proliferator-activated receptor
PL: pancreatic lipase
RG: resin glycoside
SPE: sweet potato extract
sWAT: subcutaneous white adipose tissue
TC: total cholesterol
TNF-α: tumor necrosis factor α
VAT: visceral adipose tissue.
