# Supplementary material for: Sweet potato extract alleviates high-fat-diet-induced obesity in C57BL/6J mice, but not by inhibiting pancreatic lipases
Source: Front Nutr. 2022 Nov 24;9:1016020. doi: 10.3389/fnut.2022.1016020 (PMC9731405; doi:10.3389/fnut.2022.1016020)
Supplement: Supplementary file 1 [file Data_Sheet_1.PDF]

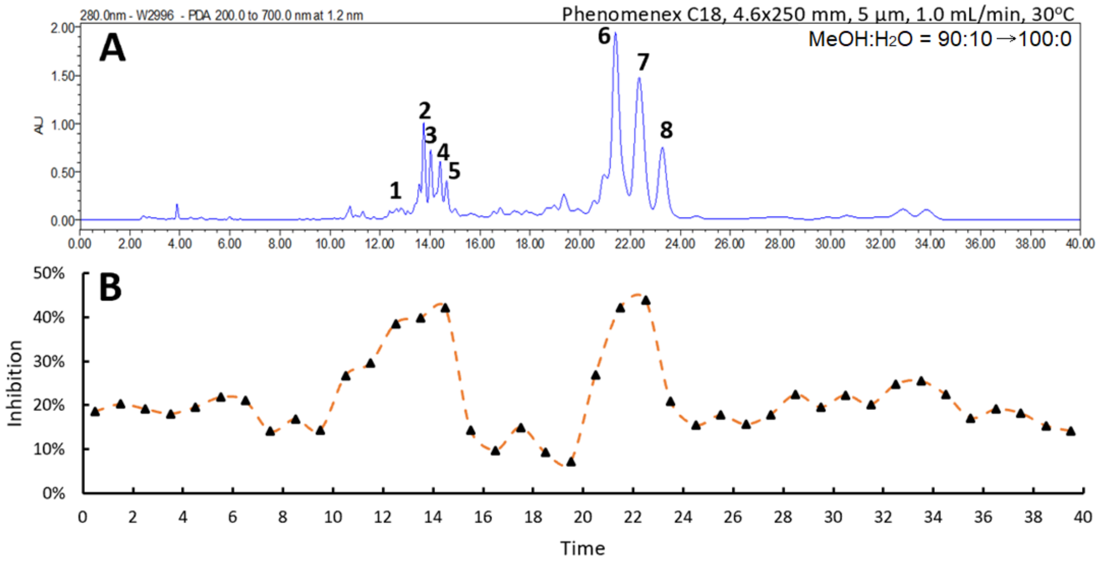

Supplementary Figure S1. HPLC analysis of fraction 5. (A) HPLC chromatogram detected at 280 nm. (B) PL inhibition of each HPLC fraction.

Supplementary Table S1. Compositions of the experimental diets.

Ingredient	Normal diet (ND)		High-fat diet (HFD)	
	gm%	kcal%	gm%	kcal%
Protein	20	20.3	26	20
Carbohydrate	64	63.9	26	20
Fat	7	15.8	35	60
kcal/gm	4		5.26	
Daily kcals consumption per mouse	11.34 ± 0.1460 (ND mice)		13.26 ± 0.2106 (HFD mice)	

Supplementary Table S2. Primer sequences for real-time PCR.

Gene	Forward (5'-3')	Reverse (5'-3')
IL-6	TAGTCCTTCCTACCCCAATTTCC	TTGGTCCTTAGCCACTCCTTC
NF-kB	GGAGGCATGTTCGGTAGTGG	CCCTGCGTTGGATTTCGTG
TNFα	GACAGTGACCTGGACTGTGG	TGAGACAGAGGCAACCTGAC
PAPRα	CAGGAGAGCAGGGATTTGCA	CCTACGCTCAGCCCTCTTCAT

CPT1	CTCAGTGGGAGCGACTCTTCA	GGCCTCTGTGGTACACGACAA
GAPDH	AGGTCGGTGTGAACGGATTTG	TGTAGACCATGTAGTTGAGGTCA

Supplementary Table S3. Observed masses and possible structure of resin glycosides in fraction 5.

Peak	t_R (min)	-MS (m/z)	$-MS^2$ (m/z)	Compound Assignment	Ref.
1	12.8	1319	1165 $[M - H - C_{10}H_{18}O]^-$, 1137 $[M - H - C_{12}H_{22}O]^-$, 1037, 983	Simonin IV	[1]
2	13.7	1267	1197 $[M - H - C_4H_6O]^-$, 1139, 1183 $[M - H - C_5H_8O]^-$, 1069, 1017	Batataoside I/II / Batatoside A	[2, 3]
3	14.0	1267	1197 $[M - H - C_4H_6O]^-$, 1139, 1183 $[M - H - C_5H_8O]^-$, 1039, 1017		
4	14.4	1281	1197 $[M - H - C_5H_8O]^-$, 1153, 963, 417	Batatoside III / Pescaprein XXVII	[4, 5]
5	14.7	1281	1197 $[M - H - C_5H_8O]^-$, 1153, 1067, 981		
6	21.4	1379	1295 $[M - H - C_5H_8O]^-$, 1251, 1197 $[M - H - C_{12}H_{22}O]^-$, 1085, 417	Batatinoside I / Batatoside C/D/E/F	[2, 6]
7	22.4	1379	1295 $[M - H - C_5H_8O]^-$, 1250, 1197 $[M - H - C_{12}H_{22}O]^-$, 831, 417		
8	23.4	1395	1311 $[M - H - C_5H_8O]^-$, 1247, 1213 $[M - H - C_{12}H_{22}O]^-$, 1065, 417	Batatoside H/I	[7]

References for Supplementary Table S3:

1. Noda, N., et al., *Resin glycosides. XV. Simonins IV, ether-soluble resin glycosides (jalapins) from the roots of Ipomoea batatas (cv. Simon).* Chemical and Pharmaceutical bulletin, 1992. **40**(12): p. 3163-3168.
2. Yin, Y., Y. Li, and L. Kong, *Pentasaccharide glycosides from the tubers of sweet potato (Ipomoea batatas).* Journal of agricultural and food chemistry, 2008. **56**(7): p. 2363-2368.
3. Yin, Y.-Q. and L.-Y. Kong, *Ether-soluble resin glycosides from the roots of Ipomoea batatas.* Journal of Asian natural products research, 2008. **10**(3): p. 233-238.
4. Yin, Y.-Q., et al., *Three new pentasaccharide resin glycosides from the roots of sweet potato (Ipomoea batatas).* Chemical and Pharmaceutical Bulletin, 2008. **56**(12): p. 1670-1674.
5. Yu, B.-W., et al., *Pentasaccharide resin glycosides from Ipomoea pes-caprae.* Journal of natural products, 2011. **74**(4): p. 620-628.
6. Escalante-Sánchez, E. and R. Pereda-Miranda, *Batatins I and II, ester-type dimers of acylated pentasaccharides from the resin glycosides of sweet potato.* Journal of natural products, 2007. **70**(6): p. 1029-1034.
7. Yin, Y.-Q., et al., *Novel acylated lipo-oligosaccharides from the tubers of Ipomoea batatas.* Carbohydrate research, 2009. **344**(4): p. 466-473.
